# Association between childhood maltreatment and suicidal ideation among Chinese patients with chronic schizophrenia: the mediating role of insomnia

**DOI:** 10.1192/bjo.2024.36

**Published:** 2024-05-03

**Authors:** Yuzhu Hao, Pu Peng, Qianjin Wang, Yanan Zhou, Shubao Chen, Qiuxia Wu, Tieqiao Liu, Xiangyang Zhang

**Affiliations:** Department of Psychiatry, National Clinical Research Centre for Mental Disorders, and National Centre for Mental Disorders, The Second Xiangya Hospital of Central South University, Changsha, China; Department of Psychiatry, Hunan Brain Hospital (Hunan Second People's Hospital), Changsha, China; CAS Key Laboratory of Mental Health, Institute of Psychology, Chinese Academy of Sciences, Beijing, China; Department of Psychology, University of Chinese Academy of Sciences, Beijing, China

**Keywords:** Schizophrenia, insomnia, suicide, childhood maltreatment, mediating effect

## Abstract

**Background:**

Childhood maltreatment is a well-established transdiagnostic risk factor for suicidal ideation; however, previous studies on their association in schizophrenia have produced highly varied results. Moreover, the mechanism linking childhood maltreatment and suicide ideation remains unclear in schizophrenia.

**Aims:**

This cross-sectional study aimed to investigate the association between childhood maltreatment and suicide ideation in people with schizophrenia and tested whether insomnia mediated this relationship.

**Method:**

Positive and Negative Syndrome Scale (PANSS), Insomnia Severity Index (ISI), Childhood Trauma Questionnaire – Short Form and Beck Suicidal Ideation Inventory were employed. Logistic regression and mediation analysis were performed.

**Results:**

(a) The prevalence of suicide ideation, insomnia, sexual abuse, emotional neglect, emotional abuse, physical abuse and physical neglect was 10% (*n* = 61), 18% (*n* = 111), 11% (*n* = 68), 25% (*n* = 153), 6.3% (*n* = 39), 17% (*n* = 106) and 39% (*n* = 239), respectively. In all, 52% (*n* = 320) reported childhood maltreatment; (b) patients with suicide ideation demonstrated higher insomnia and childhood maltreatment. PANSS depression factor, ISI, lifetime suicidal attempts and emotional abuse were independently associated with suicide ideation; (c) insomnia partially mediated the effects of emotional abuse and emotional neglect on suicide ideation, and insomnia completely mediated the effects of physical neglect and physical abuse on suicide ideation.

**Conclusion:**

Our study calls for formal assessments for childhood maltreatment and insomnia in schizophrenia, which might help identify suicide ideation early. In addition, interventions targeting insomnia might help reduce suicide ideation among people with schizophrenia who experience childhood maltreatment.

Schizophrenia could be one of the most disabling mental disorders. It affects 0.28% of the global population and is highly associated with premature mortality.^1^ Suicide ranks as the primary cause of mortality among individuals with schizophrenia.^2^ A recent meta-analysis confirms that people with schizophrenia are ten times more likely to die by suicide than the healthy population.^2^ Suicidal ideation is the earliest stage of the suicide continuum, which may evolve into suicide plans, then potentially lead to suicide attempts and ultimately result in completed suicide.^3^ The global lifetime prevalence of suicide ideation in people with schizophrenia is 34.5%.^4^ The high suicidal risk highlights the strong need for uncovering the risk factors and mechanism of suicide ideation in individuals with schizophrenia, which facilitates early identification and targeted interventions for suicide in people with schizophrenia.

## Association between childhood maltreatment and suicidality in people with schizophrenia

Childhood maltreatment is a widely recognised risk factor for suicide ideation that transcends diagnostic boundaries.^5^ However, the association between childhood maltreatment and suicide ideation in people with schizophrenia remains inconclusive. First, previous studies have produced highly varied results.^6–13^ Several studies reported no association between childhood maltreatment and suicide ideation in people with chronic schizophrenia^10^ or people with first-episode schizophrenia,^6^ while other studies demonstrated a positive relationship.^7,11^

Second, previous research has identified varying associations between specific types of childhood maltreatment, i.e. emotional abuse, emotional neglect, physical neglect, physical abuse and sexual abuse, with suicidality in the general population.^14,15^ Abuse, particularly emotional abuse and sexual abuse, have been found to be stronger predictors of suicidality compared to neglect.^14,15^ This distinction could stem from their more pronounced links with depression, emotional response and control issues.^16,17^ However, the connection between suicidality and specific types of childhood maltreatment in individuals with schizophrenia remains debatable. For example, while physical neglect is highlighted as the sole predictor for suicide ideation in some studies,^11,13^ one separate study in 216 Chinese people with schizophrenia demonstrated that suicide ideation was only positively correlated with emotional abuse and emotional neglect, but not with physical neglect.^12^ Another study in individuals with relapse schizophrenia demonstrated that suicide ideation could be related to emotional neglect and sexual abuse, while suicide ideation was not associated with other types of childhood maltreatment.^6^ The different sample characteristics and relatively small sample sizes (*N* = 50–313) in previous studies might contribute to these inconsistencies.

More importantly, the mechanism linking suicide ideation and childhood maltreatment remains largely unknown in schizophrenia. Only one study suggested that negative schema and rumination could be a mediator between childhood maltreatment and suicide ideation among people with early psychosis.^7^ Taken together, a large-scale study is warranted to generate more robust evidence of the association between childhood maltreatment and suicide ideation in people with schizophrenia and to elucidate potential mechanisms.

## Insomnia as a potential mediator between childhood maltreatment and suicide ideation

Insomnia is a common and perilous comorbidity in schizophrenia.^18^ It is closely associated with both childhood maltreatment ^19^ and higher suicide ideation in individuals with schizophrenia.^20^ Emerging studies suggest that insomnia might serve as a potential mediator linking childhood maltreatment and suicide ideation. First, longitudinal studies have shown that the predictive role of childhood maltreatment in later-life insomnia and the predictive role of insomnia in subsequent suicide ideation, which provided preliminary evidence for the theorised time-ordered effect of the mediated relationships from childhood maltreatment to insomnia to suicide ideation. Second, theoretically, according to the narrative crisis model of suicide,^21^ childhood maltreatment serves as a distal factor for suicide, a long-term vulnerability that could facilitate suicide ideation, while insomnia acts as a proximal factor, a short-term risk factor potentially precipitating an imminent suicide risk.^5^ The role of distal factors in shaping suicidal risk could be partially or fully mediated by proximal factors.^5^ Third, biological evidence also supported the potential mediating role of insomnia. Research indicates that childhood maltreatment can lead to a complex interplay between the hypothalamic-pituitary-adrenal (HPA) axis and sleep regulation mechanisms. This dynamic interaction often results in a bidirectional relationship where sleep disturbances and HPA axis dysfunction mutually exacerbate each other,^22^ leading to higher suicidal risk.^23,24^ Finally, previous empirical studies have already found that insomnia could mediate the relationship between childhood maltreatment and suicide ideation in adolescents with depression ^25^ and the non-clinical population.^5^ A recent large-scale study in people with schizophrenia-spectrum disorders also found that the impact of childhood maltreatment on clinical symptoms was mediated through insomnia.^19^ However, whether insomnia could mediate the relationship between childhood maltreatment and suicide ideation in individuals with schizophrenia remained largely undiscovered, which motivated us to conduct the present study.

## Current study

Therefore, the present study aimed to (a) determine the prevalence of childhood maltreatment and its subtypes, insomnia and suicide ideation among a large sample of Chinese people with schizophrenia; (b) evaluate the association between insomnia, childhood maltreatment and suicide ideation, including the mediating role of insomnia between childhood maltreatment and suicide ideation. Our hypotheses are that: (a) people with schizophrenia with suicide ideation will exhibit higher levels of childhood maltreatment and insomnia; (b) abuse will have a stronger association with suicide ideation compared to neglect; and (c) the association between childhood maltreatment and suicide ideation may be mediated by insomnia in schizophrenia.

## Method

### Participants and study procedure

This cross-sectional study was conducted in 2019. In-patients with chronic schizophrenia were recruited from the following psychiatric hospitals in China: Chaohu Hospital of Anhui Medical University, The Third People's Hospital of Changshu, The Third People's Hospital of Ganzhou, The Affiliated Brain Hospital of Guangzhou Medical University, Hebei Province Veterans Hospital, Mental Health Centre of Jingzhou, Nanning Fifth People's Hospital, Ning An Hospital of Ningxia, Shanghai Pudong New Area Mental Health Centre, Shanghai Putuo District Mental Health Centre, Shandong Mental Health Centre, Shanxi Province Veterans Hospital, Shenyang Anning Hospital, Suzhou Guangji Hospital, Wenzhou Kangning Hospital, Wuhan Mental Health Centre and The Third Affiliated Hospital of Sun Yat-sen University. They provide mental-health and other medical services to patients. The inclusion criteria included: (a) a diagnosis of schizophrenia confirmed by trained psychiatrists using the Structured Clinical Interview for Diagnostic and Statistical Manual of Mental Disorders, fourth edition (SCID-DSM-IV); (b) an illness duration of more than 1 year; (c) a stable clinical status, with medication stability (regular antipsychotic treatment for at least 6 months) and symptoms stability (symptoms did not significantly improve or worsen, and no new symptoms emerged within the last 2 weeks before the study entry); and (d) Han Chinese population, age 18–70 years. The exclusion criteria included: (a) comorbidity with several physical illnesses (such as severe central nervous system diseases; acute, unstable or life-threatening medical illnesses such as cancer, and ongoing infections) or any other major mental disorder confirmed by SCID; (b) pregnancy or breastfeeding; (c) comorbidity with substance use disorder (excluding tobacco).

The assessments included three steps. First, participants were interviewed face to face by two trained psychiatrists using SCID to assess their eligibility based on the inclusion and exclusion criteria. Second, the positive and negative syndrome scale (PANSS) was administered to each participant by these two psychiatrists who had previously received training on the rating of PANSS, and the inter-rater correlation coefficient of PANSS scores exceeded 0.8. Finally, participants were given self-reported questionnaires (Childhood Trauma Questionnaire – Short Form [CTQ-SF]; Insomnia Severity Index [ISI]; Beck Scale for Suicidal Ideation [BSSI]) in hard copy to complete independently. For those unable to read simplified Chinese, the psychiatrists read out the items and options. Psychiatrists were available throughout to clarify any questions or concerns, ensuring participants fully understood the questionnaire. The assessments were conducted in quiet, private spaces within the hospital to maintain confidentiality and minimise distractions.

### Sample size calculation

The determination of our sample size was guided by several considerations: (a) Prevalence estimates: the formula for calculating sample size is: *n* = [*Z*^2^*p*(1−*p*)]/*E*^2^ (*n*, sample size; *Z*, Z-score corresponding to the desired confidence level; *p*: expected incidence rate; *E*, allowable error margin). For suicide ideation, we utilised an estimated prevalence of 18% in Chinese patients with schizophrenia,^26^ allowing a 5% error margin. This calculation yielded a required sample size of 252. In the case of childhood maltreatment, with an estimated prevalence of 81.5%,^27^ the resulting necessary sample size was 236. In the case of insomnia, with an estimated prevalence of 18%,^28^ the resulting necessary sample size was 246. (b) Logistic regression model: following recent guidelines,^29^ a minimum sample size of 500 is deemed essential for obtaining statistically reliable results in logistic regression analyses. (c) Mediation analysis: to detect a ‘small’ indirect effect in mediation analysis using the bootstrapping method, a minimum of 560 participants is needed.^30^ Taken together, the targeted sample size was 560. However, to enhance the robustness of our results, we adopted a continuous and ongoing sampling strategy, aiming to recruit as many participants as possible.

### Measurements

#### Basic information

The following demographic and clinical variables were collected from self-designed questionnaires: age, gender, education years, smoking status (current smoker/ex-smoker/non-smoker) and marital status (single/married). The following variables were collected from medical records: body mass index (BMI), family history of psychiatric disorders, age at onset and duration of schizophrenia, history of hypertension and diabetes, and the use of antipsychotic drugs. The daily dose of antipsychotic medication was converted to an equivalent dose of chlorpromazine.

#### Clinical symptoms

Two trained psychiatrists independently evaluated the psychotic symptoms of each participant using PANSS. PANSS is a 30-item, clinician-rated, 7-point Likert scale, measuring psychotic symptoms within the last week. It comprises three subscales: the positive symptom subscale (items P1–7), negative symptom subscale (items N1–7) and general psychopathology subscale (items G1–16).^31^ Following the previous study,^32^ the PANSS depressive factor (G2, G3, G6) and PANSS cognitive factor (P2, N5, G11) were calculated to determine depressive and cognitive symptoms. The Chinese version of PANSS has demonstrated excellent reliability and validity, and has been widely utilised among Chinese people with schizophrenia.^33^ In our study, the inter-rater correlation coefficient for the PANSS total score exceeded 0.8, and Cronbach's α of the PANSS was 0.916.

#### Childhood trauma

We employed CTQ-SF to retrospectively measure childhood trauma occurring before the age of 18.^34^CTQ-SF consists of 28 items and encompasses five subscales: childhood emotional abuse, emotional neglect, physical abuse, physical neglect and sexual abuse.^35^ The CTQ-SF has demonstrated excellent psychometric properties within the Chinese population and has been widely utilised among patients with schizophrenia.^35^ The scores of each subscale of CTQ-SF ranged from 5 to 25, with higher scores indicating more severe childhood trauma. Following previous psychometric evidence,^34^ we utilised the following cut-off points for each CTQ-SF subscale to identify the presence of childhood trauma: emotional abuse ≥ 13, physical abuse ≥ 10, sexual abuse ≥ 8, emotional neglect ≥ 15 and physical neglect ≥ 10. The Cronbach's α of CTQ-SF was 0.723.

#### Insomnia

ISI measured insomnia,^36^ with ISI being a validated self-reported questionnaire for recent insomnia over the previous two weeks. ISI included seven items, with total scores ranging from 0 to 21. Following previous psychometric evidence,^37^ participants with ISI scores over 7 were classified as the insomnia group. The Cronbach's α of ISI was 0.912.

#### Suicidal ideation

We used BSSI to measure current suicide ideation within the previous week,^38^ and the Chinese version showed acceptable reliability.^39^ BSSI is composed of 19 items, with higher scores indicating more severe suicide ideation. As suggested by Beck et al,^40^ a cut-off point of 3 for BSSI was used to identify the current suicide ideation. We also measured the history of lifetime suicidal attempts through face-to-face interviews and medical records. A single question (‘Have you ever attempted to kill yourself in your lifetime?’) was delivered to all participants.^41^ Those who responded ‘Yes’ were further questioned about the method, time and frequency of suicidal attempts. We contacted their family members when necessary.

### Statistical analysis

Continuous data were presented as mean and standard deviation (s.d.), while categorical data were reported as frequency and percentage. First, we compared demographic and clinical variables (insomnia, childhood maltreatment and clinical symptoms) between patients with and without current suicide ideation. Chi-square tests and student t-tests were utilised as appropriate. Cohen's *d* was calculated to determine the effect size of the association of childhood maltreatment, insomnia and clinical symptoms on suicide ideation, with values below 0.5 indicating a small effect, between 0.5 and 0.8 indicating a medium effect and above 0.8 indicating a large effect.

Second, we performed a stepwise logistic regression analysis to identify correlated predictors of the current suicide ideation. Variables that demonstrated statistical significance (*P* < 0.05) in the univariate analysis were included.

Third, we conducted receiver operating characteristic (ROC) analysis and calculated the area under the curve (AUC) values to assess the discriminatory performance of our model in distinguishing participants with and without suicide ideation. AUC values of 0.7–0.79 were considered acceptable, 0.8–0.89 excellent and ≥0.9 outstanding.^42^

Finally, we conducted mediation analysis to examine the hypothesis that childhood trauma could impact suicide ideation through insomnia. Hence, we set childhood trauma (CTQ, sexual abuse, emotional neglect, emotional abuse, physical abuse or physical neglect) as independent variables, insomnia as mediator variable and suicide ideation as dependent variable. The following demographic (age, gender, education years, married status, BMI and smoking) and clinical characteristics (history of suicidal attempts, age at onset, family history of schizophrenia, total chlorpromazine (CPZ) dosages and PANSS scores) were controlled as covariates in the mediation analysis. We employed Model 4 in the PROCESS procedure of Statistical Package for the Social Sciences (SPSS) version 27.0. Bootstrap sampling was performed 5000 times to generate 95% CI. All statistical analyses were conducted using SPSS. The tests were two-tailed, and statistical significance was set at *P* < 0.05. The ROC curve was plotted using R 4.3.0 (http://www.R-project.org/).

## Results

### Sample characteristics

In total, 702 people with schizophrenia were approached and screened. Eighty-four were excluded because of missing data on CTQ, PANSS or BSSI, resulting in a final sample size of 618. The majority of the participants were male (67%), single (78%) and non-smokers (58%). The mean age, age at onset and illness duration for the participants were 43, 25 and 18 years, respectively.

A total of 10 and 15% of the participants reported current suicide ideation and lifetime suicidal attempts, respectively. The prevalence of insomnia, sexual abuse, emotional neglect, emotional abuse, physical abuse and physical neglect was 18%, 11%, 25%, 6.3%, 17%, and 39%, respectively. In all, 52% (*n* = 320) of the participants reported at least one type of childhood trauma.

We obtained complete medication records for 90.3% of participants, and their usage of antipsychotic drugs was summarised as the follows: clozapine (*n* = 211), risperidone (*n* = 325), olanzapine (*n* = 135), aripiprazole (*n* = 128), quetiapine (*n* = 76), sulpiride (*n* = 16), amisulpride (*n* = 43), perphenazine (*n* = 13), ziprasidone (*n* = 19), chlorpromazine (*n* = 39) and others (*n* = 5). A total of 265 (47%) of the participants were prescribed multiple antipsychotic drugs. The mean daily dose of the antipsychotics was equivalent to 432 mg/day of CPZ.

### Comparison in basic and clinical information between patients with and without suicide ideation

As shown in Table 1, individuals with suicide ideation were younger and had shorter illness durations compared to those without. We observed no difference between participants with and without suicide ideation regarding other basic information.

**Table 1:** Sample characteristics of patients with and without suicidal ideation

Variable	Overall *N* = 618^a^	Without suicidal ideation, *N* = 557^a^	With suicidal ideation, *N* = 61^a^	*P*-value^b^	Cohen's *d*
Gender				0.2	
Male	415 (67%)	378 (68%)	37 (61%)		
Female	202 (33%)	178 (32%)	24 (39%)		
Age, years	43 (13)	43 (13)	39 (12)	**0.010**	
Education, years	9.3 (3.0)	9.2 (3.0)	9.6 (3.1)	0.3	
Marriage				0.6	
Single	481 (78%)	435 (78%)	46 (75%)		
Married	136 (22%)	121 (22%)	15 (25%)		
History of diabetes	75 (12%)	69 (12%)	6 (9.8%)	0.6	
History of hypertension	22 (3.6%)	20 (3.6%)	2 (3.3%)	>0.9	
Age at onset, years	25 (8)	25 (8)	24 (8)	0.8	
Illness duration, years	18 (12)	19 (12)	14 (11)	**0.006**	
Smoking status				0.7	
Non-smoker	358 (58%)	326 (59%)	32 (52%)		
Ex-smoker	72 (12%)	64 (11%)	8 (13%)		
Current smoker	188 (30%)	167 (30%)	21 (34%)		
Family history of psychosis	114 (18%)	99 (18%)	15 (25%)	0.2	
Antipsychotic drug use	432 (267)	437 (269)	391 (240)	0.2	
BMI, kg/m^2^	24.3 (4.9)	24.3 (4.9)	24.1 (4.9)	0.8	
History of suicidal attempts	90 (15%)	68 (12%)	22 (36%)	**<0**.**001**	
PANSS-positive subscale	17 (7)	17 (7)	21 (6)	**<0**.**001**	0.48
PANSS-negative subscale	23 (8)	23 (8)	24 (7)	0.8	0.03
PANSS-general psychopathology subscale	38 (12)	38 (12)	43 (11)	**<0**.**001**	0.48
PANSS depression factor	5.78 (2.94)	5.49 (2.67)	8.46 (3.88)	**<0**.**001**	1.06
PANSS cognitive factor	7.76 (2.79)	7.74 (2.82)	7.93 (2.52)	0.6	0.07
PANSS total scores	79 (23)	78 (23)	87 (20)	**0**.**001**	0.42
CTQ-EA	7.14 (2.88)	6.92 (2.69)	9.08 (3.70)	**<0**.**001**	0.77
Exposure to emotional abuse	39 (6.3%)	27 (4.8%)	12 (20%)	**<0**.**001**	
CTQ-PA	6.23 (2.53)	6.08 (2.35)	7.62 (3.54)	**0**.**001**	0.62
Exposure to physical abuse	106 (17%)	85 (15%)	21 (34%)	**<0**.**001**	
CTQ-SA	5.74 (2.01)	5.67 (1.94)	6.41 (2.51)	**0**.**029**	
Exposure to sexual abuse	68 (11%)	55 (9.9%)	13 (21%)	**0**.**007**	0.37
CTQ-EN	11.6 (4.8)	11.5 (4.8)	13.1 (4.6)	**0**.**011**	
Exposure to emotional neglect	153 (25%)	129 (23%)	24 (39%)	**0**.**005**	0.34
CTQ-PN	8.92 (3.10)	8.76 (3.06)	10.38 (3.12)	**<0**.**001**	
Exposure to physical neglect	239 (39%)	200 (36%)	39 (64%)	**<0**.**001**	0.53
CTQ-SF total scores	40 (11)	39 (10)	47 (12)	**<0**.**001**	0.72
At least one childhood maltreatment	320 (52%)	273 (49%)	47 (77%)	**<0**.**001**	
ISI	3.4 (4.5)	3.1 (4.3)	6.3 (5.6)	**<0**.**001**	0.72
Insomnia	111 (18%)	86 (15%)	25 (41%)	**<0**.**001**	

a. *n* (%); Mean (s.d.).

b. Pearson's Chi-squared test; t-test.

PANSS, Positive and Negative Syndrome Scale; CTQ-EA, Childhood Trauma Questionnaire – Emotional Abuse subscale; CTQ-PA, Childhood Trauma Questionnaire – Physical Abuse subscale; CTQ-SA, Childhood Trauma Questionnaire – Sexual Abuse subscale; CTQ-EN, Childhood Trauma Questionnaire – Emotional Neglect subscale; CTQ-PN, Childhood Trauma Questionnaire – Physical Neglect subscale;CTQ-SF, Childhood Trauma Questionnaire – Short Form; ISI, Insomnia Severity Index; BMI, body mass index. Results in bold show those that are significant at *p*<0.05.

Individuals with current suicide ideation graded worse in PANSS positive subscale, PANSS general psychopathology subscale and PANSS depression factor. However, no difference in PANSS negative subscale and PANSS cognitive factor was observed. They scored higher on ISI and CTQ-SF total scores as well as on all its subscales (all *P* < 0.05). The effect size was large for PANSS depression factor and medium to large for ISI, CTQ-SF total scores and CTQ-EA subscales as suggested by the Cohen *d* value. The prevalence of insomnia (41% versus 15%), lifetime history of suicidal attempts (36% versus 12%), exposure to emotional abuse (20% versus 4.8%), exposure to physical abuse (34% versus 15%), exposure to sexual abuse (21% versus 9.9%), exposure to emotional neglect (39% versus 23%), and exposure to physical neglect (64% versus 36%) were much higher in participants with suicide ideation (all *P* < 0.05).

### Correlated predictors of suicide ideation

**Fig. 1:**
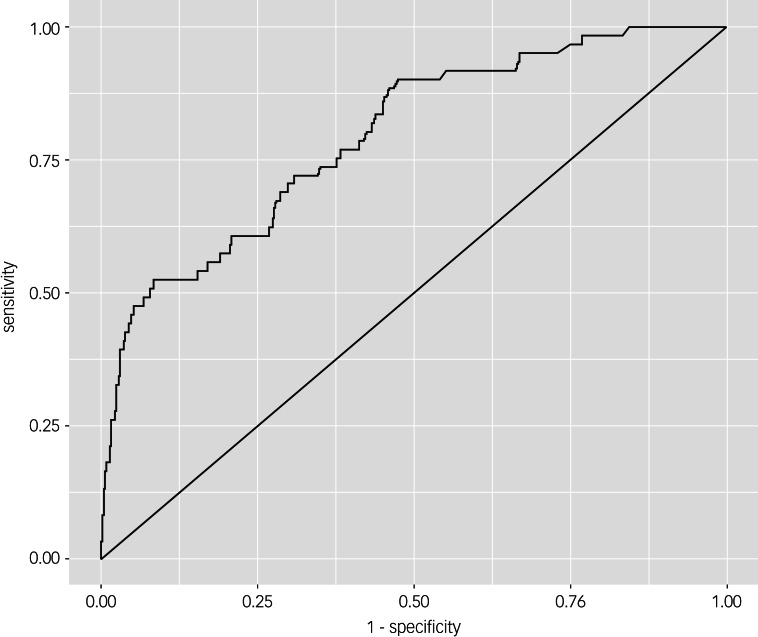
The discriminatory capacity of the logistic regression model for distinguishing between patients with and without suicide ideation. Our model demonstrated excellent discriminatory capacity in differentiating between schizophrenia patients with and without suicide ideation, with an area under the curve value of 0.80.

Table 2 displayed the results of the logistic regression model for the current suicide ideation, with the following variables being selected as independent variables: age, illness duration, PANSS depression factor, PANSS positive subscales, PANSS general psychopathology subscales, lifetime history of suicidal attempts, ISI scores, CTQ-CTQ-emotional abuse subscale, CTQ-physical neglect subscale, CTQ-physical abuse subscale, CTQ-sexual abuse subscale and CTQ-emotional neglect subscale. Finally, the lifetime history of suicidal attempts (odds ratio, 2.85; 95% CI, 1.48–5.49, *P* = 0.002), PANSS depressive factor (odds ratio, 1.24; 95% CI, 1.13–1.36, *P* < 0.001), ISI scores (odds ratio, 1.06; 95% CI, 1.01–1.13, *P* = 0.026) and CTQ-emotional abuse subscale (odds ratio, 1.18, 95% CI, 1.08–1.28, *P* < 0.001) were independently associated with current suicide ideation. Our regression model performed well in distinguishing between patients with and without suicide ideation (Fig. 1), with an AUC value of 0.80.

**Table 2:** Correlated predictors of current suicidal ideation (*n* = 609)

Variable	Odds ratio	Lower	Upper	*p*
Lifetime suicidal attempts	2.84	1.04	5.49	0.002
PANSS depressive factor	1.24	1.13	1.36	<0.001
ISI	1.06	1.01	1.13	0.027
CTQ-EA	1.18	1.08	1.28	<0.001

PANSS, Positive And Negative Syndrome Scale; ISI, Insomnia Severity Index; CTQ-EA, Childhood Trauma Questionnaire – Emotional Abuse subscale.

### Insomnia mediated the association between childhood trauma and suicide ideation

**Fig. 2:**
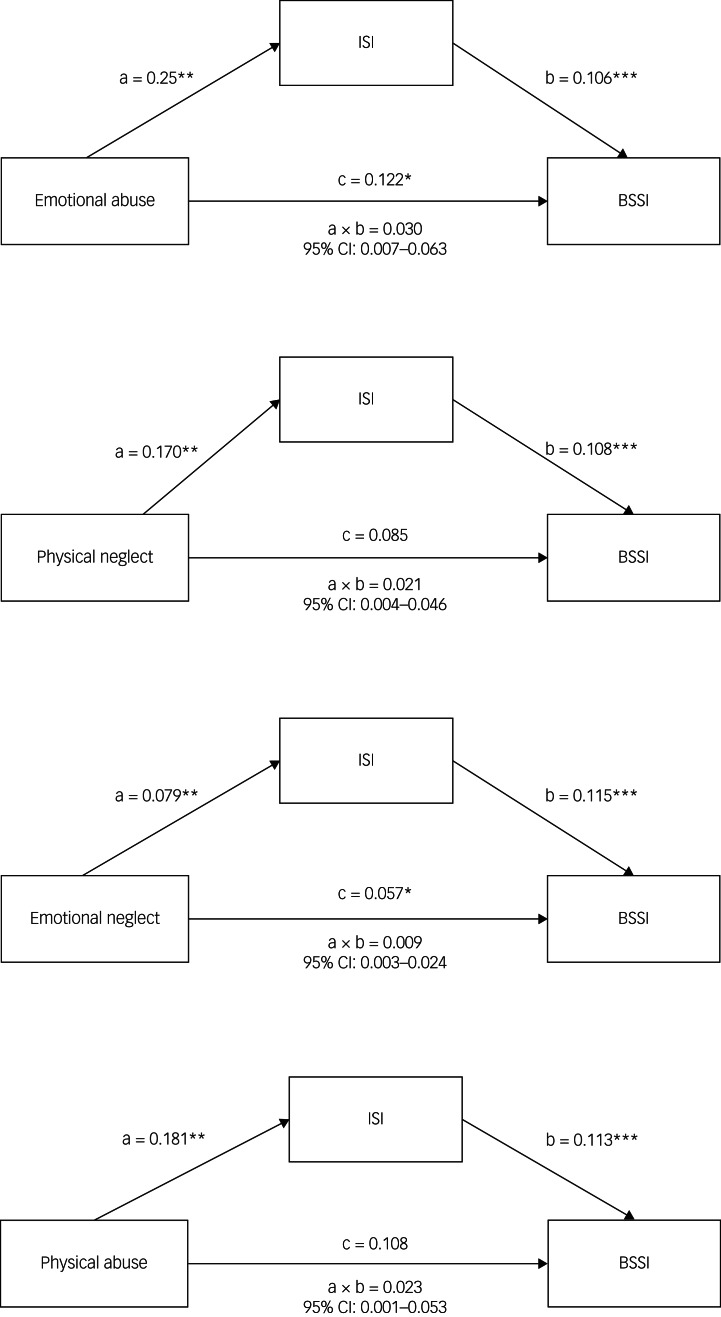
Mediating effects of insomnia on the relationship between childhood maltreatment and suicidal ideation in schizophrenia. Path a is independent variable (X) → ISI score (M). Path b is ISI score (M) → BSSI score (Y), adjusted for X. Path c is X → BSSI score (Y), adjusted for ISI score (M). Path a × b is X → Y through M. CTQ-SF, Childhood Trauma Questionnaire – Short Form; BSSI, Beck Suicidal Ideation Inventory; ISI, Insomnia Severity Index.

Table 3 and Fig. 2 describe the mediation effects of insomnia on the association between childhood trauma and suicide ideation. The 95% bootstrapped CI of the indirect effect of emotional abuse and emotional neglect on suicide ideation via insomnia did not include zero, and the direct effect was significant, indicating insomnia partially mediated their association. The 95% bootstrapped CI of the indirect effect of physical abuse and physical neglect on suicide ideation via insomnia did not include zero, but the direct effect was not significant, indicating insomnia completely mediated their relationship. However, no mediation effect of insomnia on CTQ and sexual abuse was observed.

**Table 3:** Mediation analysis: direct and indirect effects of childhood trauma on suicide ideation, taking insomnia as the mediator (*n* = 532)

	CTQ-ISI		Total effect		Direct effect		Indirect effect	
	B	95% CI	B	95% CI	B	95% CI	B	Corrected 95% CI
**CTQ-SF**	0.018	−0.0170 to 0.053	−0.002	−0.027 to 0.025	−0.004	−0.023 to 0.022	0.002	−0.003 to 0.009
**Emotional abuse**	**0**.025**	**0.126**–**0.381**	**0**.158**	**0.057**–**0.247**	**0**.122*	**0.026**–**0.218**	**0**.**030**	**0.007**–**0.063**
**Physical neglect**	**0**.170**	**0.051**–**0.290**	**0**.106*	**0.017**–**0.195**	0.085	−0.004 to 0.173	**0**.**021**	**0.004**–**0.046**
Sexual abuse	**0**.203*	**0.020**–**0.387**	0.075	−0.062 to 0.211	0.048	−0.087 to 0.183	0.026	−0.004 to 0.074
Emotional neglect	**0**.079*	**0.002**–**0.155**	**0**.067*	**0.010**–**0.123**	**0**.057*	**0.007**–**0.112**	**0**.**009**	**0.003**–**0.024**
Physical abuse	**0**.181*	**0.034**–**0.328**	**0**.130*	**0.021**–**0.239**	0.108	−0.001 to 0.216	**0**.**023**	**0.001**–**0.053**

CTQ-SF, Childhood Trauma Questionnaire – Short Form; ISI, Insomnia Severity Index.

*P < 0.05, **P < 0.01. Results in bold show those that are significant at *p*<0.05.

## Discussion

This study is the largest to date assessing the link between childhood maltreatment, insomnia and suicide ideation in Chinese individuals with schizophrenia. It for the first time reveals insomnia's mediating role between suicide ideation and childhood maltreatment, and identifies emotional abuse, insomnia, depressive symptoms and suicidal attempt history as suicide ideation predictors. The results suggested that regular screening for insomnia and childhood maltreatment were essential in people with schizophrenia, which might help early suicide ideation detection. Besides, targeting insomnia might reduce suicide ideation in patients with childhood maltreatment experiences.

### Prevalence of childhood maltreatment, insomnia and suicide ideation in patients with schizophrenia

To our knowledge, our study was the largest study of its kind to assess the prevalence of childhood maltreatment and its association with insomnia and suicide ideation in Chinese people with schizophrenia. We found approximately half of the participants reported childhood maltreatment. Neglect (physical neglect and emotional neglect) emerged as the most prevalent forms of childhood maltreatment among our participants. The prevalence of childhood maltreatment aligned with previous research conducted among both Chinese patients with schizophrenia,^12,35,43^ and patients from Western countries,^19,44^ which ranged from 47.2 to 80%. It was also much higher than those found in non-clinical samples in the Chinese population.^12,45^

Moreover, the prevalence of current suicide ideation (10%) and insomnia (18%) in our study was fairly close to the findings reported by Miller et al,^28^ who examined a cohort of 328 Chinese patients with chronic schizophrenia (12% for suicide ideation and 18% for insomnia). However, the prevalence of suicide ideation was much lower than what has been reported in Western countries.^4^ Various factors may contribute to this inconsistency: the widespread use of clozapine among Chinese people with schizophrenia and the greater stigma surrounding suicide within the Chinese cultural context may decrease the prevalence of suicide ideation, and the difference between suicide-related polygenes among Chinese and Caucasian populations may also be the reason for the lower incidence.^46^ However, the potential interactions or confounding effects of these factors on the associations between childhood maltreatment, suicide ideation and insomnia remains unclear.

### Association between suicide ideation and childhood maltreatment in patients with schizophrenia

Consistent with previous studies in the general population and people with depression,^5,47^ our study demonstrated a significant positive association between all subtypes of childhood maltreatment and increased suicide ideation. Notably, our study demonstrated that emotional abuse could be a crucial vulnerability to current suicide ideation, independent of the severity of psychiatric symptoms. These results align with a recent study conducted by Diago et al,^8^ which also identified emotional abuse as the strongest predictor of suicide ideation in individuals with first-episode psychosis. Moreover, meta-analyses have consistently shown that emotional abuse displayed the most substantial impact on suicidal attempts^48^ and non-suicidal self-injury.^49^

There are several potential explanations for the robust association between emotional abuse and suicide ideation. First, the Interpersonal Theory of Suicide proposes that suicide ideation originates from feelings of thwarted belongingness and perceived burdensomeness,^50^ both of which are closely linked to emotional abuse. Second, emotional abuse contributes to negative self-perceptions, maladaptive coping styles and impaired emotional regulation, thereby increasing vulnerability to depression, hopelessness and subsequently suicide ideation.^51^ Another possible explanation involves dysregulation of the HPA axis. Studies suggest that childhood maltreatment serves as a significant distal factor in HPA axis dysregulation, which is associated with higher suicide ideation.^52,53^ However, it is important to note that most of the existing evidence on the mechanisms linking emotional abuse and suicide ideation originates from non-clinical samples or individuals with other mental disorders. Further research is warranted to validate these hypotheses in schizophrenia.

The results of this study also indicated that higher PANSS depressive factor scores were independently associated with increased suicide ideation in patients with schizophrenia. This result is consistent with former studies that have identified the positive association between depressive symptoms and suicide ideation.^54,55^ Research has indicated that individuals with depressive symptoms often experience challenges in social functioning and have lower quality of life, which may consequently elevate the risk of suicide ideation.^56^

### Insomnia mediated the relationship between childhood maltreatment and suicide ideation

Consistent with prior findings,^18^ our studies highlighted that insomnia was independently associated with suicide ideation in patients with chronic schizophrenia. Childhood maltreatment was positively associated with insomnia. Furthermore, the mediation analysis indicated that insomnia mediated the association between physical neglect, emotional abuse and suicide ideation, which replicated findings in patients with depression.^25^ While it is well established that childhood maltreatment could lead to insomnia through traumatic stress pathways,^57^ very limited numbers of studies shed light on their association in the context of schizophrenia. One study of 418 patients with schizophrenia spectrum disorder demonstrated that insomnia could mediate the relationship between childhood maltreatment and positive symptoms, mood symptoms and poorer functioning in schizophrenia.^19^ Our results supported their hypothesis, that insomnia following childhood maltreatment could further exacerbate worse clinical status in schizophrenia. However, conflicted results were also reported. For instance, another study involving 155 people with schizophrenia found no mediation effect of insomnia on the relationship between traumatic experiences and positive symptoms.^58^ Given the lack of existing literature on the association between childhood maltreatment, insomnia and clinical symptoms in schizophrenia, further large-scale studies are needed to verify our findings and explore the potential biological mechanism.

### Implications of our findings

Our study holds two significant clinical implications. First, our study demonstrated that emotional abuse and insomnia served as predictors for current suicide ideation, independently of the demographic information and the severity of psychotic symptoms. Therefore, regular assessments of insomnia and childhood maltreatment are essential in schizophrenia to detect suicide ideation. Particular attention should be paid to patients with emotional abuse and insomnia. Second, our study highlights the mediating role of insomnia in the association between childhood maltreatment and suicide ideation. Therefore, targeting insomnia in individuals with schizophrenia with childhood maltreatment might be helpful in reducing childhood maltreatment-related suicide ideation.

### Limitations

Several limitations should be acknowledged. First, the cross-sectional study design prevented us from establishing causal relationships. The results of mediation were preliminary. Further longitudinal studies are essential to confirm the mediating role of insomnia in the relationship between childhood maltreatment and suicide ideation in schizophrenia. Second, childhood maltreatment, insomnia and suicide ideation were assessed through self-reported questionnaires, which might induce recall and social desirability bias. Furthermore, the study revealed that individuals exhibiting escalated mental health symptoms might recall childhood experiences through a negative lens, potentially leading to additional bias in retrospective self-report measures of childhood maltreatment.^59^ Third, we did not collect information about sleep duration, which is closely associated with insomnia but not measured by ISI. Fourth, the study's cohort consisted solely of Chinese individuals with chronic schizophrenia, excluding those with complex comorbidities. Therefore, the generalisability of our findings to broader populations, such as outpatients, international populations and those with comorbid conditions, necessitates further studies to confirm. Fifth, several important psychological and biological factors such as resilience, coping style, cortisol and inflammation levels associated with childhood maltreatment, insomnia and suicide ideation were not measured.^23,24^ Taken together, a further longitudinal study with more comprehensive assessments and participants with schizophrenia of different clinical status or cultural backgrounds is warranted to confirm our findings.

To conclude, our study provides new insights into the complicated relationship between childhood maltreatment, insomnia and suicide ideation in schizophrenia. Our study suggested that childhood maltreatment and insomnia were prevalent among people with schizophrenia. Depressive symptoms, insomnia, lifetime history of suicidal attempts and emotional abuse were independently associated with current suicide ideation. Hence, formal and regular assessments for childhood maltreatment and insomnia are vital in this population. Particular attention should be paid to suicide ideation among patients with emotional abuse and insomnia. In addition, insomnia partially or totally mediated the relationship between childhood maltreatment and suicide ideation. Thus, interventions targeting insomnia might hold promise for reducing suicide ideation among individuals with schizophrenia who have experienced childhood maltreatment.

## Supporting information

Hao et al. supplementary material Hao et al. supplementary material

## Data Availability

The data are available from the corresponding author on reasonable request.
