# Supplementary material for: Association between childhood maltreatment and suicidal ideation among Chinese patients with chronic schizophrenia: the mediating role of insomnia
Source: BJPsych Open. 2024 May 3;10(3):e98. doi: 10.1192/bjo.2024.36 (PMC11094451; doi:10.1192/bjo.2024.36)

| **Variable** | **Overall**, N = 618^1^ | **Without SI**, N = 557^1^ | **With SI**, N = 61^1^ | **p-value**^2^ | **Cohen D** |
| --- | --- | --- | --- | --- | --- |
| **gender** |  |  |  | 0.2 |  |
| Male | 415 (67%) | 378 (68%) | 37 (61%) |  |  |
| Female | 202 (33%) | 178 (32%) | 24 (39%) |  |  |
| **age** | 42 (33, 53) | 43 (33, 54) | 40 (31, 47) | 0.022 |  |
| **Education years** | 8.0 (8.0, 11.0) | 8.0 (8.0, 11.0) | 9.0 (8.0, 11.0) | 0.2 |  |
| **marriage** |  |  |  | 0.6 |  |
| Single | 481 (78%) | 435 (78%) | 46 (75%) |  |  |
| Married | 136 (22%) | 121 (22%) | 15 (25%) |  |  |
| **Diabetes** | 75 (12%) | 69 (12%) | 6 (9.8%) | 0.6 |  |
| **Hypertension** | 22 (3.6%) | 20 (3.6%) | 2 (3.3%) | >0.9 |  |
| **Age at onset** | 23 (19, 28) | 23 (19, 28) | 22 (18, 29) | 0.8 |  |
| **Family history** | 114 (18%) | 99 (18%) | 15 (25%) | 0.2 |  |
| **CPZ total dose** | 375 (240, 600) | 380 (240, 600) | 338 (214, 572) | 0.3 |  |
| **BMI, kg/m^2^** | 23.8 (21.2, 26.9) | 23.7 (21.3, 26.8) | 23.9 (20.4, 27.3) | 0.7 |  |
| **Smoking status** |  |  |  | 0.7 |  |
| Non-smoker | 358 (58%) | 326 (59%) | 32 (52%) |  |  |
| EX-smoker | 72 (12%) | 64 (11%) | 8 (13%) |  |  |
| Current-smoker | 188 (30%) | 167 (30%) | 21 (34%) |  |  |
| **EA** | 6 (5, 9) | 6 (5, 8) | 8 (6, 11) | <0.001 | 0.77 |
| **PA** | 5 (5, 6) | 5 (5, 6) | 6 (5, 8) | <0.001 | 0.62 |
| **SA** | 5 (5, 5) | 5 (5, 5) | 5 (5, 7) | 0.002 | 0.37 |
| **EN** | 11 (8, 14) | 10 (8, 14) | 13 (10, 16) | 0.006 | 0.34 |
| **PN** | 9 (6, 11) | 8 (6, 11) | 10 (8, 13) | <0.001 | 0.53 |
| **ISI** | 1 (0, 5) | 1 (0, 5) | 6 (1, 11) | <0.001 | 0.72 |
| **PANSS-Positive** | 17 (11, 23) | 16 (11, 22) | 20 (17, 25) | <0.001 | 0.48 |
| **PANSS-Negative** | 24 (18, 29) | 24 (18, 29) | 23 (20, 29) | 0.9 | 0.03 |
| **PANSS-General** | 37 (29, 47) | 37 (28, 46) | 43 (36, 50) | <0.001 | 0.48 |
| **PANSS-Depression** | 5 (3, 8) | 5 (3, 7) | 8 (6, 11) | <0.001 | 1.06 |
| **PANSS-Cognitive** | 8 (6, 10) | 8 (6, 10) | 7 (7, 10) | 0.6 | 0.07 |
| **PANSS total scores** | 78 (62, 97) | 77 (61, 96) | 87 (75, 99) | 0.002 | 0.42 |
| **History of suicidal attempts** | 90 (15%) | 68 (12%) | 22 (36%) | <0.001 |  |
| **SA8** | 68 (11%) | 55 (9.9%) | 13 (21%) | 0.007 |  |
| **EN15** | 153 (25%) | 129 (23%) | 24 (39%) | 0.005 |  |
| **EA13** | 39 (6.3%) | 27 (4.8%) | 12 (20%) | <0.001 |  |
| **PA8** | 106 (17%) | 85 (15%) | 21 (34%) | <0.001 |  |
| **PN10** | 239 (39%) | 200 (36%) | 39 (64%) | <0.001 |  |
| **CTQ** | 320 (52%) | 273 (49%) | 47 (77%) | <0.001 |  |
| ^1^n (%); Median (IQR) | | | | |  |
| ^2^Pearson's Chi-squared test; Wilcoxon rank sum test; Fisher's exact test | | | | |  |

Table 2 Independent correlates of current SI

| Variables | OR | lower | upper | p |
| --- | --- | --- | --- | --- |
| Lifetime suicidal attempts | 3.13 | 1.60 | 6.12 | <0.001 |
| PANSS depressive factor | 1.24 | 1.13 | 1.36 | <0.001 |
| Insomnia | 2.20 | 1.15 | 4.20 | 0.017 |
| EA | 2.54 | 1.03 | 6.27 | 0.043 |
| PN | 2.47 | 1.32 | 4.60 | 0.005 |

Note:

Table 3 Mediation analysis: direct and indirect effects of childhood trauma on SI, taking insomnia as the mediator

|  | CT-ISI | | Total effect | | Direct effect | | Indirect effect | | Proportion mediated % |
| --- | --- | --- | --- | --- | --- | --- | --- | --- | --- |
|  | B | 95%CI | B | 95%CI | B | 95%CI | B | Corrected 95%CI |  |
| **CTQ** | **0.052**** | **0.190-0.085** | **0.047***** | **0.023-0.070** | **0.040***** | **0.017-0.064** | **0.006** | **0.001-0.014** | **12.8%** |
| **EA** | **0.208***** | **0.084-0.333** | **0.189***** | **0.101-0.278** | **0.165**** | **0.077-0.254** | **0.024** | **0.006-0.053** | **12.7%** |
| **PN** | **0.129*** | **0.012-0.245** | **0.106*** | **0.023-0.173** | **0.090*** | **0.008-0.173** | **0.016** | **0.001-0.039** | **15.1%** |
| SA | 0.163 | -0.014-0.341 | 0.101 | -0.026-0.228 | 0.080 | -0.046-0.206 | 0.022 | -0.006-0.065 | NA |
| EN | 0.069 | -0.005-0.143 | **0.064*** | **0.011-0.117** | **0.055*** | **0.003-0.108** | 0.009 | -0.001-0.024 | NA |
| PA | 0.133 | -0.008-0.274 | **0.149**** | **0.049-0.250** | **0.133**** | **0.034-0.233** | 0.017 | -0.022-0.044 | NA |


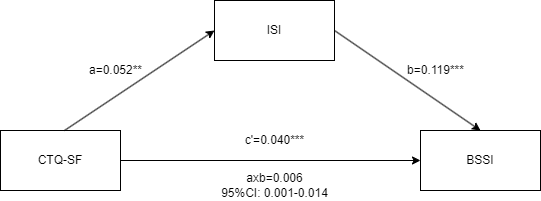

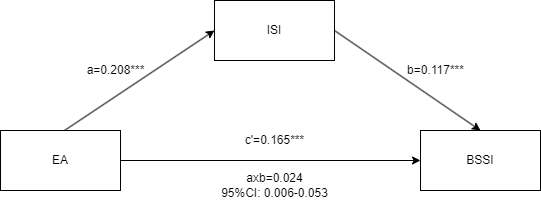

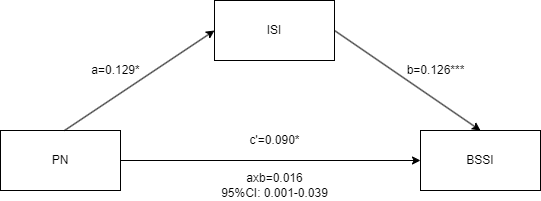

Supplement: Hao et al. supplementary material [file S205647242400036Xsup001.docx]
